# A Tick Gut Protein with Fibronectin III Domains Aids *Borrelia burgdorferi* Congregation to the Gut during Transmission

**DOI:** 10.1371/journal.ppat.1004278

**Published:** 2014-08-07

**Authors:** Sukanya Narasimhan, Jeroen Coumou, Tim J. Schuijt, Eric Boder, Joppe W. Hovius, Erol Fikrig

**Affiliations:** 1 Department of Internal Medicine, Yale University School of Medicine, New Haven, Connecticut, United States of America; 2 Center for Experimental and Molecular Medicine, Academic Medical Center, University of Amsterdam, Amsterdam, The Netherlands; 3 Department of Chemical and Biomolecular of Engineering, University of Tennessee, Knoxville, Tennessee, United States of America; 4 Howard Hughes Medical Institute, Chevy Chase, Maryland, United States of America; Oklahoma State University, United States of America

## Abstract

*Borrelia burgdorferi* transmission to the vertebrate host commences with growth of the spirochete in the tick gut and migration from the gut to the salivary glands. This complex process, involving intimate interactions of the spirochete with the gut epithelium, is pivotal to transmission. We utilized a yeast surface display library of tick gut proteins to perform a global screen for tick gut proteins that might interact with *Borrelia* membrane proteins. A putative fibronectin type III domain-containing tick gut protein (Ixofin3D) was most frequently identified from this screen and prioritized for further analysis. Immunization against Ixofin3D and RNA interference-mediated reduction in expression of Ixofin3D resulted in decreased spirochete burden in tick salivary glands and in the murine host. Microscopic examination showed decreased aggregation of spirochetes on the gut epithelium concomitant with reduced expression of Ixofin3D. Our observations suggest that the interaction between *Borrelia* and Ixofin3D facilitates spirochete congregation to the gut during transmission, and provides a “molecular exit” direction for spirochete egress from the gut.

## Author Summary

Lyme borreliosis, the most common vector-borne illness in Northeastern parts of USA, is caused by *Borrelia burgdorferi* sensu lato spirochetes, and transmitted by the *Ixodes scapularis* ticks. Currently there is no vaccine available to prevent Lyme borreliosis. A better understanding of tick proteins that interact with *Borrelia* to facilitate spirochete transmission could identify new targets for the development of a tick-based vaccine to prevent Lyme borreliosis. Spirochete growth and exit from the gut is central to transmission, and might involve intimate interactions between the spirochete and the tick gut. We therefore performed a global screen to identify *Borrelia*-interacting tick gut proteins. One of the four *Borrelia*-interacting tick proteins, referred to as Ixofin3D, was further characterized. RNA-interference-mediated down-regulation of Ixofin3D resulted in decreased spirochete numbers in the salivary glands and consequently decreased transmission to the host during tick feeding. We demonstrate that Ixofin3D aids spirochete congregation to the gut epithelium, a critical first step that might direct spirochete exit from the gut.

## Introduction

*Ixodes scapularis* is the predominant vector of several human pathogens including *Borrelia burgdorferi*, the agent of Lyme borreliosis in North America [1], [2]. There is currently no commercial vaccine to prevent Lyme borreliosis in humans [3], although recent efforts have been made in that direction [4]. An increased molecular understanding of how the tick acquires, sustains and transmits *Borrelia* would be conducive to the development of novel strategies, including anti-tick vaccines [5]–[7], to control Lyme borreliosis. *Borrelia* resides in the unfed tick gut anchored to a gut protein, TROSPA [8]. Transmission begins with the growth of the spirochetes in the gut when the *Borrelia*-infected tick begins to take a blood meal. Somewhere between 24 and 36 hours of tick feeding [9], coincident perhaps with an optimal quorum of spirochetes or its gene expression profile [10], [11], the spirochete travels from the gut to the salivary glands from where it exits the tick along with tick saliva into the host skin. Growth and migration from the tick gut is therefore an essential prelude to *Borrelia* transmission.

The spirochete proteome changes dramatically during tick feeding to facilitate migration from the gut [10]. Rudenko *et al* [12] showed that *Borrelia* infection alters the transcriptome of the *Ixodes ricinus* gut during feeding, suggesting a dynamic interaction between the tick gut and the growing spirochete. Consistent with this, Dunham-Ems *et al* [13] showed by live imaging of spirochetes in feeding *I. scapularis* guts that the spirochete engages intimately with the epithelial cells of the tick gut during transmission, moving away from the gut lumen towards the basal lamina of the gut. It is likely that *Borrelia*-gut interactions provide molecular signals that direct the movement of the spirochete from the luminal side of the gut epithelium to the basal lamina of the gut to facilitate egress from the gut. Further, Zhang *et al* [14] showed that interaction between a secreted tick gut protein, TRE31, and a spirochete outer surface protein BBE31 enables migration of the spirochete through the hemolymph to the salivary glands by mechanisms that remain to be understood. These observations highlight a thematic strategy of the spirochete to interact with gut proteins during growth and migration, which we are only just beginning to understand. In order to delineate the complex molecular interactions of spirochetes with the gut epithelium we screened for *Borrelia*-interacting tick gut proteins by probing a tick gut yeast surface display (YSD) library with *Borrelia* outer surface proteins. The YSD approach has traditionally utilized specific proteins individually to probe libraries of single chain antibodies to identify and characterize protein-protein interactions [15]. Work by Cho and Shusta [16] demonstrated that biotinylated whole cell lysates of mammalian cell lines or plasma membrane proteins can be used to screen a YSD library expressing human single chain antibody fragments and identify specific antigen-antibody interactions without *a priori* knowledge of the candidate antigens [17]. Building on this work, we have, in this study, extended the utility of YSD to examine tick gut-*B. burgdorferi* interactions without *a priori* knowledge of either interactants.

We screened 10^7^ tick gut YSD clones with total *Borrelia* membrane extracts derived from *in vitro*-grown *B. burgdorferi* N40 and identified four potential *Borrelia*-interacting gut proteins from the initial screen. One of the predominant clones encoded a surface exposed tick gut protein with four putative fibronectin type III domains. In this report, we present our observations that suggest that the fibronectin type III domain-containing tick gut protein helps congregation of spirochetes to the gut epithelium during transmission. These observations invoke a functional role for spirochete “clustering” in spirochete egress from the gut.

## Results

### Yeast surface display library screening identifies four potential *Borrelia*-interacting tick gut proteins

**Figure 1:**
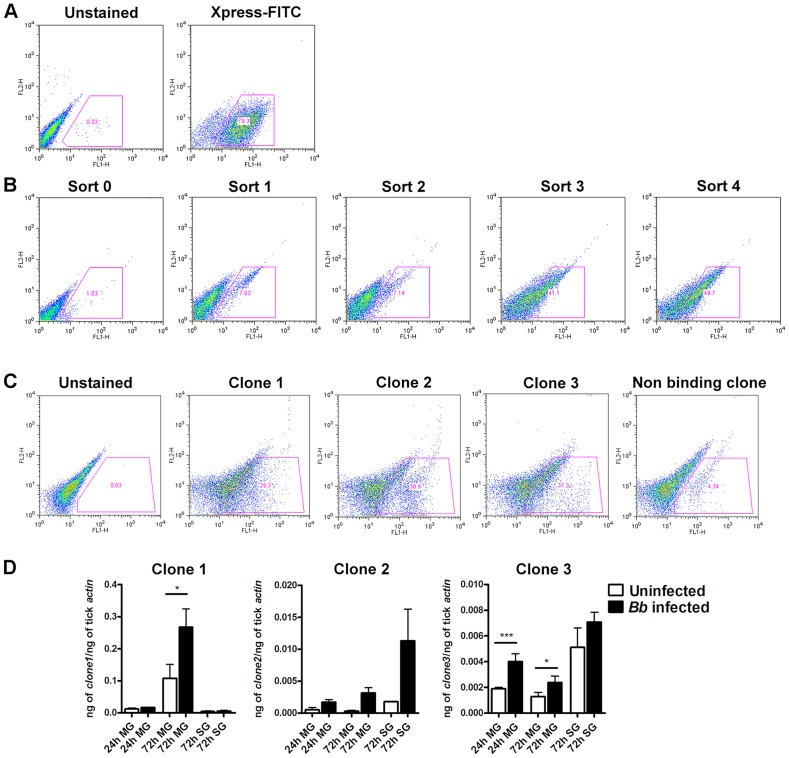
Yeast Surface Display (YSD) approach to identify tick gut proteins that interact with *B. burgdorferi* membrane proteins. **A.** EBY-100 yeast cells transformed with an *Ixodes scapularis* salivary gland cDNA library were induced overnight before magnetic sorting. The following day, surface tick protein expression as fusion proteins with the yeast agglutinin protein Aga2p was confirmed with antibodies against the Xpress epitope using flow cytometry. **B.** After each magnetic sort, binding of Alexa488-labeled *B. burgdorferi* membrane protein extract to EBY-100 yeast cells was analysed using flow cytometry. **C.** Flow cytometric analysis of individual yeast displayed clones from Sort 4. *Borrelia*-binding clones 1, 2 and 3 and one representative non-binding clones are shown. In panels **B** and **C** cell populations within the gate represent yeast-displayed clones bound to Alexa488-labeled *B. burgdorferi* membrane protein extract. **D.** Quantitative RT-PCR assessment of the mRNA expression profiles of the genes encoding clones 1, 2 and 3 in salivary glands (SG) and guts (MG) of *B. burgdorferi*-infected and uninfected nymphs at 24 and 72 hours post tick attachment. Error bars represent mean ± SEM and mean values significantly different in a two-tailed non-parametric Mann-Whitney test (*p*<0.05) indicated by one asterisk (*p<0.05*) or two asterisks (*p*<0.01).

*B. burgdorferi* membrane protein extracts were prepared as described [18] from *in vitro* grown *B. burgdorferi* (N40) temperature-shifted to 37°C for 24 hours. A YSD expression library of *I. scapularis* gut cDNAs [14] was probed with biotin-labeled *B. burgdorferi* membrane protein extracts as described in Materials and Methods. Four rounds of magnetic-activated cell sorting (MACS) screens provided a 40-fold enrichment of YSD clones expressing gut proteins that interacted with *B. burgdorferi* membrane proteins (Fig. 1A–B). Cells from the 4^th^ sort were plated and one hundred colonies were individually tested for their ability to bind to *B. burgdorferi* membrane protein extracts by fluorescence-activated cell sorting (FACS) analysis using Alexa488-labeled *B. burgdorferi* membrane protein extracts. Recombinant plasmids were isolated from colonies that showed greater than 15% binding (40 clones) (Fig. 1C) and insert sizes compared by restriction digestion analysis. Clones with identical insert sizes were grouped (four groups) and two representative clones from each group were sequenced. Four unique clones encoding partial fragments of tick gut proteins were identified and provided a unique identifier based on their *in silico* predicted function (Table 1).

**Table 1:** *I. scapularis* gut yeast surface display library screening identifies four potential *Borrelia*-interacting tick gut proteins.

Clone	Number of times identified in the screen	% cells binding to Bb membrane proteins	Gene identity^I^	Matching region on ORF^I^	Possibly missing exons^II^	Putative function and domainsIII	Cellular location^IV^	Signal peptide^V^	Trans-membrane domainIII	MW^VI^ (kDa)	Paralogs^I^	Orthologs^I^
**1**	15	29%	ISCW008121	294–1347	Yes	Cell-adhesion (fibronectin III domains)	Transmembrane	Yes	Yes	65*	Yes (3)	None
**2**	10	30%	ISCW015135	1–320	No	None	Extracellular	Yes	None	13	None	None
**3**	10	37%	ISCW015049	1–604	Yes	Cell-adhesion (Dystrophin-associated glycoprotein-1)	Uncertain	None	Yes	91	None	Yes (8)
**4**	3	26%	ISCW016197	192–762	Yes	Guanylate-kinase associated protein (GKAP)	Nuclear	None	None	70	None	Yes (13)

I database of www.vectorbase.org,

II as stated on Genbank annotations,

III SMART domain prediction (http://smart.embl-heidelberg.de),

IV PSORT (http://psort.hgc.jp/),

V SignalP analysis (www.cbs.dtu.dk/services/SignalP/),

VI Theoretical molecular weight (MW) in kDa using ExPASy proteomics server (http://web.expasy.org/compute_pi/).

**Figure 2:**
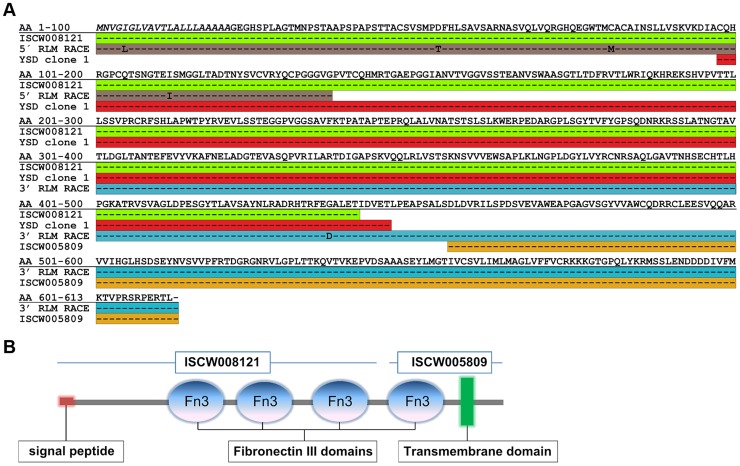
Full-length sequence of Ixofin3D (Genbank accession number KF709698). **A.** Amino acid (AA) sequence corresponding to: the annotated ISCW008121 (green), fragment obtained by 5′ RNA Ligase Mediated Rapid Amplification of cDNA Ends (RLM-RACE) (grey), yeast surface display Clone 1 (red), fragment obtained by 3′ RLM-RACE (blue), and the annotated ISCW005809 (yellow). **B**. Predicted analysis of full-length Ixofin3D using the Simple Modular Architecture Research Tool available at http://smart.embl-heidelberg.de.

*Based on Ixofin3D sequence, see Figure 2. ORF: Open Reading Frame.

Clone 1 (identified 15 times in this screen, ∼29% binding) contained an in-frame insert that showed 100% identity to ISCW008121 (www.vectorbase.org) and encoded a protein that contained four putative fibronectin type III-domains. PSORT (http://psort.hgc.jp/) protein localization analysis suggested that it was likely a surface exposed transmembrane protein (Table 1). Clone 2 (identified 10 times in this screen, ∼30% binding) showed 100% identity to ISCW015135. ISCW015135 encoded a putative signal peptide indicative of a secreted protein as seen by Signal P analysis (www.cbs.dtu.dk/services/SignalP). No known functions or domains were identified, and no paralogs or orthologs of Clone 2 were observed by BLAST analysis. Clone 3 (identified 10 times in this screen, ∼37% binding) showed 100% identity to ISCW015049 and encoded a protein with two dystroglycan-like cadherin domains. The cellular location could not be predicted by PSORT analysis. Orthologs of clone 3 were found in eight other invertebrate vectors. Clone 4 (identified three times, 26% binding) showed 100% identity to ISCW016197 and encoded a nuclear membrane localization signal and a putative Guanylate-kinase associated protein domain, and orthologs were identified in 13 other invertebrate vectors (Table 1). *B. burgdorferi* is an extracellular pathogen, hence the physiological relevance of interactions between *B. burgdorferi* extracellular proteins and a tick nuclear protein (clone 4) is unclear and hence not prioritized for further analysis.

The expression profiles of the genes contained in the three prioritized clones were assessed by quantitative RT-PCR (qRT-PCR) in the salivary glands and guts of *I. scapularis* nymphs (Fig. 1D) during feeding. While ISCW008121 (Clone 1) was expressed preferentially in the gut, ISCW015135 and ISCW015049 (clones 2 and 3) were expressed both in the salivary glands and guts. Furthermore, *B. burgdorferi* infection increased the expressions of Clones 1 and 3 significantly (∼2-fold) in the guts after 72 hours of tick feeding (Fig. 1D).

### Full-length ISCW008121 encodes a protein with four putative fibronectin type III domains and a transmembrane domain

We addressed Clone 1 in further detail because it was identified most frequently in this screen and because Clone 1 encoded a tick protein with fibronectin type III domains. While *B. burgdorferi* has been shown to encode lipoproteins that facilitate *Borrelia* adhesion to fibronectin in the mammalian host to promote infection [19], it is not known if similar interactions occur in the tick. Further, fibronectin type III domains, originally identified in the extracellular matrix protein fibronectin [20], have also been identified in several receptor-like proteins and shown to play a critical role in cell signaling [21]. Therefore, we reasoned that understanding the physiological significance of Clone 1-*Borrelia* interaction might provide new insights into tick-*Borrelia* interactions.

The protein encoded by YSD Clone 1 in this screen encompasses amino acids 97 to 449 of the protein encoded by ISCW008121 (Figure 2A). To confirm the start site of the annotated ISCW008121 (www.vectorbase.org), we performed a 5′end RLM RACE (RNA Ligase-Mediated Rapid Amplification of cDNA Ends) using fed *I. scapularis* gut total RNA and gene specific primers complementary to the first 411 bp of the annotated ISCW008121 gene transcript. Further, YSD Clone 1 sequence and the annotated ISCW008121 transcript did not contain a stop codon, suggesting that the annotated gene sequence did not contain a full-length 3′ sequence. Therefore, we performed a 3′end RLM RACE using fed *I. scapularis* gut total RNA and gene-specific primers, and identified the transcript from bp 1350 to 1836, complete with stop codon. The full-length sequence was shown to encode a ∼66 kDa protein containing four fibronectin type III domains and a transmembrane domain (Fig. 2B) and is henceforth referred to as ***Ixo*** *des scapularis* **Fi**bro**n**ectin **3 D**omain-containing gut protein (Ixofin3D) and is assigned the GenBank accession number KF709698.

### Ixofin3D-PF binds *in vitro* grown spirochetes

**Figure 3:**
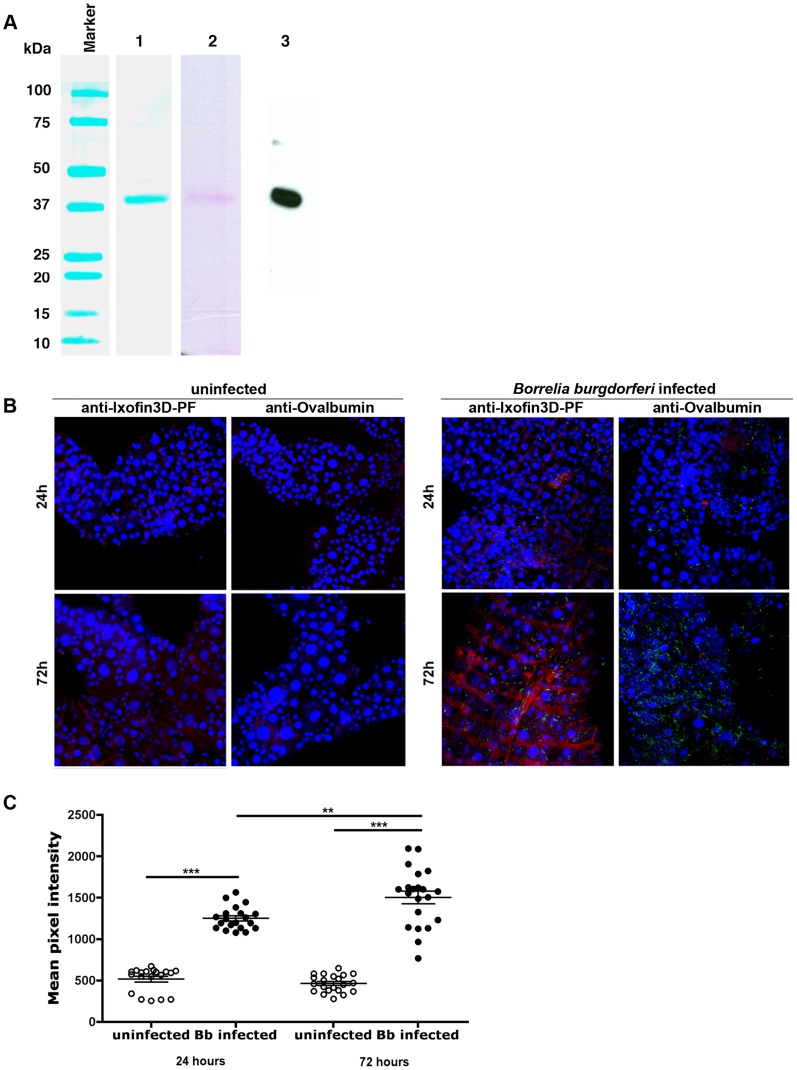
Ixofin3D localization in the tick gut. **A.** Purified *Drosophila*-expressed recombinant Ixofin3D-PF electrophoresed on SDS 12% polyacrylamide gel and **Lane 1**, Coomassie blue stained; **Lane 2**, Periodic Acid-Schiff stained; and **Lane 3**, rIxofin3D-PF immunoblotted and probed with polyclonal rabbit anti-Ixofin3D-PF serum. **B**. Confocal microscopy of PFA-fixed guts of 24 and 72h fed uninfected and *B. burgdorferi*-infected nymphs. Gut nuclei, *B. burgdorferi* and Ixofin3D stained with TO-PRO-3 (blue), anti *B. burgdorferi* (FITC-green) and anti-rIxofin3D-PF serum (TRITC-red) respectively. Magnification ×20. Guts stained with anti-Ovalbumin IgG (TRITC-red) served as antibody control. **C**. Mean pixel intensities of regions of interest in the TRITC channel (representing anti-rIxofin3D-PF serum binding to Ixofin3D) of the confocal images obtained in **B**. Each data point represents one region of interest. Error bars represent mean ± SEM and mean values significantly different in a one-way ANOVA with Tukey's multiple comparison test indicated by two asterisks (*p*<0.01) or indicated by three asterisks (*p*<0.0001).

**Figure 4:**
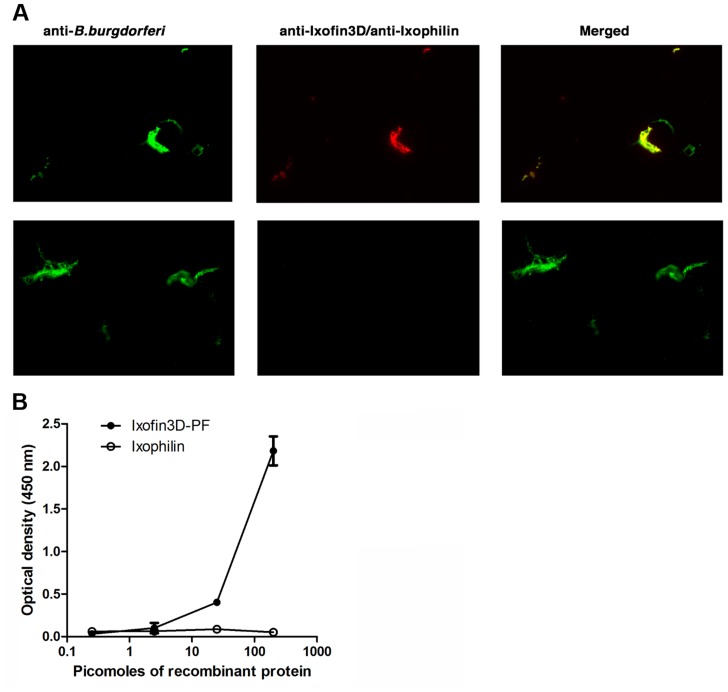
*In vitro* analysis of Ixofin3D-*Borrelia burgdorferi* interaction. **A.** Imunofluorescence microscopy of PFA-fixed *in vitro* grown *B. burgdorferi* to assess binding to rIxofin3D-PF. *B. burgdorferi* was detected with FITC-conjugated *B. burgdorferi* antisera (FITC-green), rIxofin3D-PF (Panel 1) was detected using rabbit anti-Ixofin3D-PF IgG (TRITC-red), and rIxophilin (Panel 2) was detected using mouse anti-rIxophilin IgG (TRITC-red). Magnification ×20. **B.** ELISA assessment of dose-dependent binding of rIxofin3D-PF to *B. burgdorferi* membrane protein extract-coated plates compared to rIxophilin, a tick protein that does not bind to *Borrelia*.

We were unable to express the full-length protein transcript of Ixofin3D in the *Drosophila* expression system (DES), but succeeded in expressing a **p**artial **f**ragment of Ixofin3D protein encompassing amino acids 104 to 319 (rIxofin3D-PF) that is also contained in the protein fragment expressed in YSD Clone 1 (Fig. 2A). The 37 kDa rIxofin3D-PF generated in *Drosophila* S2 cells was glycosylated as seen by Periodic-Acid Schiff's staining (Fig. 3A). Polyclonal rabbit antibodies against rIxofin3D-PF bound to uninfected and *B. burgdorferi*-infected fed guts (Fig. 3B) as seen by confocal microscopy, suggesting that Ixofin3D is expressed on the surface of the gut. Consistent with the qRT-PCR analysis, quantification of pixel intensity in the TRITC channel (representing anti-rIxofin3D-PF serum binding to native Ixofin3D) using the ImageJ software showed significantly increased binding of rIxofin3D-PF antibodies to the tick gut in 24 h and 72 h fed ticks upon *B. burgdorferi* infection compared to that in 24 h and 72 h fed uninfected guts (Fig. 3C), and in *B. burgdorferi*-infected 72 h fed tick guts when compared to *B. burgdorferi*-infected 24 h fed guts (Fig. 3C) suggesting that Ixofin3D expression was increased in *B. burgdorferi* infected guts during feeding. rIxofin3D-PF incubated with *in vitro* grown PFA-fixed non-permeabilized *B. burgdorferi* N40 showed binding of rIxofin3D-PF to spirochetes as seen by indirect immunofluorescence using rabbit polyclonal antibodies against purified rIxofin3D-PF (Fig. 4A) indicating a potential interaction between Ixofin3D and an exposed *B. burgdorferi* protein ligand. Under similar conditions, rIxophilin, a tick gut thrombin inhibitor protein [22], not known to engage directly with spirochetes, did not show binding to *in vitro* grown spirochetes (Fig. 4A). Furthermore, in an ELISA assay using microplates coated with *B. burgdorferi* membrane protein extract, a dose-dependent increase in rIxofin3D-PF binding to *B. burgdorferi* membrane protein extracts was observed, whereas dose-dependent binding of rIxophilin was not observed (Fig. 4B).

### Active and passive immunity against Ixofin3D-PF impairs *Borrelia* transmission

**Figure 5:**
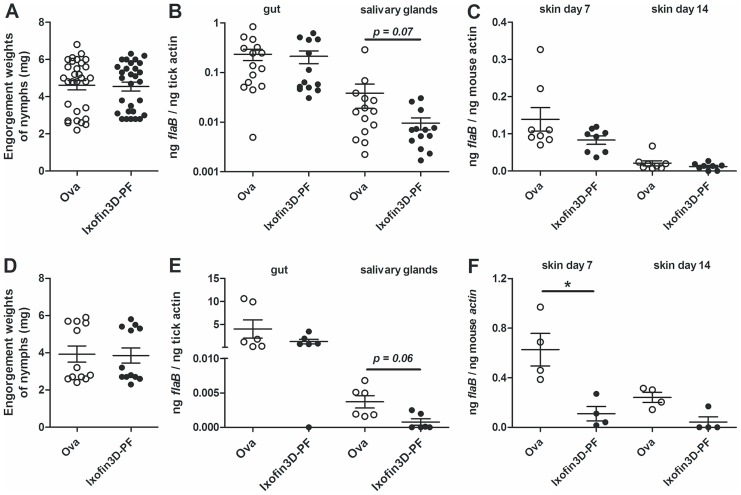
Impact of passive and active immunization against Ixofin3D-PF on *B. burgdorferi* burden in ticks and in murine skin. **A–C:** Rabbit anti-rIxofin3D-PF serum or rabbit anti-Ovalbumin serum was passively transferred into each mouse 24 h prior to *B. burgdorferi*-infected tick challenge (4–5 ticks/mouse). **A.** Engorgement weights of repleted ticks; **B.** qRT-PCR assessment of *Borrelia* burden in tick guts and salivary glands; **C.** qPCR assessment of *Borrelia* burden in murine skin at 7 and 14 days post-tick feeding. **D–F:** Mice actively immunized with rIxofin3D-PF or Ovalbumin and challenged with *B. burgdorferi*-infected ticks (4–5 ticks/mouse). **D.** Engorgement weights of repleted ticks; **E.** qRT-PCR assessment of *Borrelia* burden in tick guts and salivary glands; **F.** qPCR assessment of *Borrelia* burden in mice skin at 7 and 14 days post-tick feeding. Each data point in A and D represents one tick. Each data point in B and E represents a pool of 2–3 guts or salivary glands. Each data point in C and F represents one mouse. Error bars represent mean ± SEM and mean values significantly different in a two-tailed non-parametric Mann-Whitney test (*p*<0.05) indicated by an asterisk. A representative of three experiments is shown.

To determine the role of Ixofin3D in spirochete transmission, we passively transferred purified rabbit IgG against rIxofin3D-PF into eight C3H/HeN mice and challenged these mice with *Borrelia*-infected *I. scapularis* nymphs. Control mice received purified rabbit IgG against ovalbumin (Ova). Ticks fed to repletion and engorged comparably on both control and experimental mice (Fig. 5A). Guts and salivary glands were dissected from engorged nymphs and *Borrelia* burden assessed by qRT-PCR. While the spirochete burden in the guts were comparable in both groups, *Borrelia* burden in the salivary glands was reduced in nymphs that fed on mice that received anti-rIxofin3D-PF antibodies (Fig. 5B) when compared to that in salivary glands of nymphs fed on mice that received anti-Ovalbumin antibodies, however, the decrease was not statistically significant. *Borrelia* burden in the skin of mice that received anti-rIxofin3D-PF antibodies (Fig. 5C) was also reduced at 7 days post tick feeding when compared to that in the skin of mice that received anti-Ovalbumin antibodies, however, the decrease was not statistically significant.

As seen with passive immunization, active immunization against rIxofin3D-PF did not impact the engorgement weights of nymphal ticks, and *Borrelia* burden in the nymphal guts (Fig. 5D–E). Active immunization against rIxofin3D-PF decreased *Borrelia* burden in the salivary glands of fed nymphs, although, the decrease was not statistically significant (Fig. 5E). However, *Borrelia* burden in the skin of mice at 7 days post tick feeding was significantly reduced when compared to that in the skin of mice that were immunized against ovalbumin (Fig. 5F).

### RNA interference-mediated decrease in *ixofin3D* expression results in decreased *Borrelia* transmission

**Figure 6:**
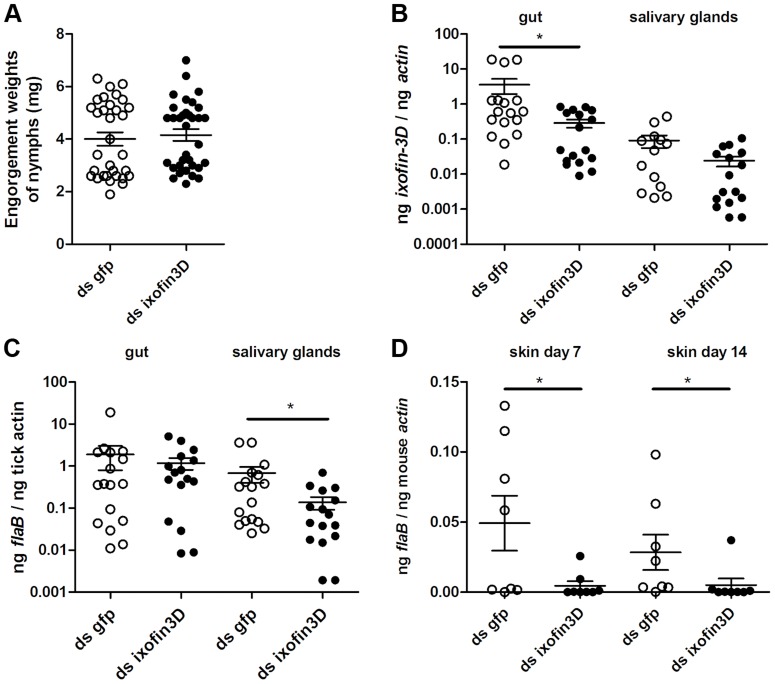
RNA interference-mediated decrease in Ixofin3D results in decreased *B. burgdorferi* burden in the salivary glands and in murine skin. Double-stranded *ixofin3D* (ds *ixofin3D*) or ds *gfp* was injected through the anal pore 3 h prior to *B. burgdorferi*-infected tick challenge (4–5 ticks/mouse). **A**. Engorgement weights of ticks fed to repletion. Each data point represents one tick; **B**. qRT-PCR assessment of *ixofin3D* expression; and **C**. *Borrelia* burden in tick guts and salivary glands. Each data point in B and C represents a pool of 3 guts or salivary glands; and **D**. qPCR assessment of *Borrelia* burden in murine skin at 7 and 14 days post-tick feeding. Each data point represents one mouse. Error bars represent mean ± SEM and mean values significantly different in a two-tailed non-parametric Mann-Whitney test (*p*<0.05) indicated by an asterisk. A representative of 3 experiments is shown.

To circumvent the possibility that antibodies against partial Ixofin3D might not efficiently abrogate Ixofin3D function *in vivo*, and to clarify the role of Ixofin3D in *Borrelia* transmission, we decreased the expression of Ixofin3D by RNA interference (RNAi) as described earlier [23]. Four to five double stranded (ds) *ixofin3D* RNA-injected nymphs or ds *gfp* RNA-injected were allowed to engorge on each mouse (8 mice/group). Nymphs injected with ds *ixofin3D* RNA engorged comparably to control nymphs injected with ds *gfp* RNA (Fig. 6A) despite a significant decrease in the expression of *ixofin3D* RNA in the guts as seen by qRT-PCR (Fig. 6B). While *Borrelia* burden in the guts was comparable in ds *gfp* and ds *ixofin3D*-injected nymphs (Fig. 6C), *Borrelia* burden in the salivary glands of fed ds *ixofin3D*-injected nymphs when compared to that in the salivary glands of ds *gfp* RNA-injected nymphs was significantly decreased (Fig. 6C). *Borrelia* burden in the skin of mice fed upon by ds *ixofin3D* RNA-injected nymphs (experimental group) at 7 and 14 days post tick feeding was significantly decreased when compared to that in the skin of mice that were fed upon by ds *gfp* RNA-injected nymphs (Fig. 6D).

### RNAi-mediated knock-down of *ixofin3D* expression results in decreased aggregation of spirochetes on the tick gut

**Figure 7:**
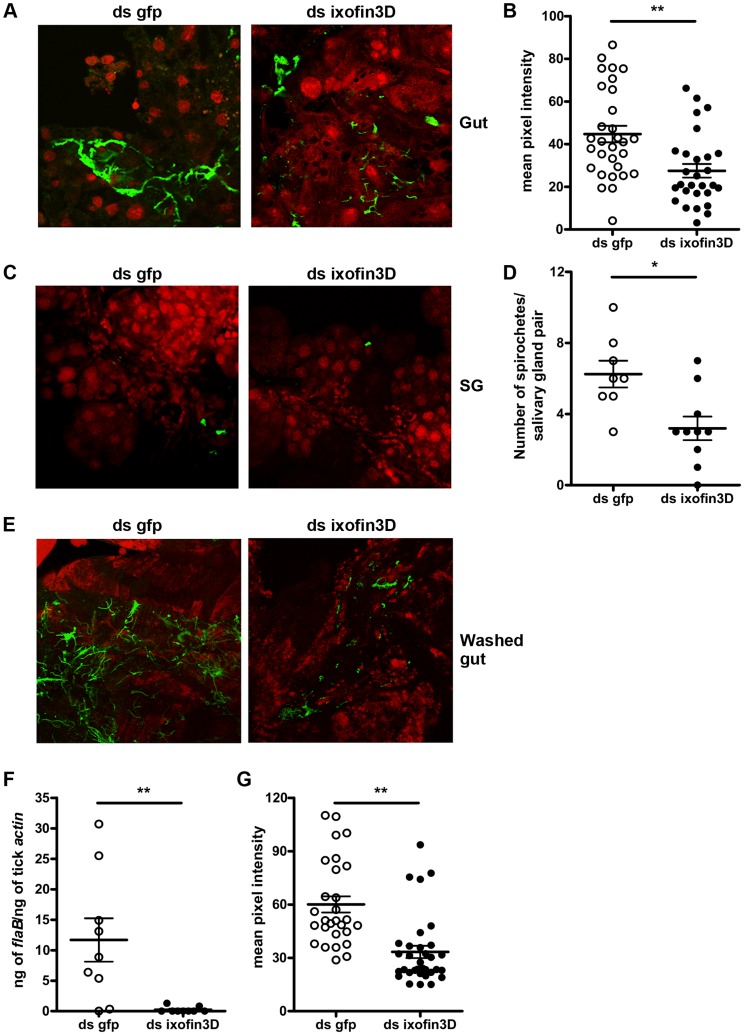
RNA-mediated knockdown of *ixofin3D* expression results in decreased aggregation of *Borrelia burgdorferi* on the gut. **A.** Confocal microscopy of PFA-fixed guts from 72 h fed *B. burgdorferi*-infected nymphs injected with ds *ixofin3D* or ds *gfp* RNA. Nuclei, and spirochetes stained with propidium iodide (red), and anti-*B. burgdorferi* (N40) IgG (FITC-green) respectively. **B**. Mean pixel intensities of regions of interest in the FITC channel (representing anti-*B. burgdorferi* serum binding to spirochetes) of the confocal images obtained in **A**. Each data point represents one region of interest. **C**. Confocal microscopy of PFA-fixed salivary glands from 72 h fed *B. burgdorferi*-infected nymphs injected with ds *ixofin3D* or ds *gfp* RNA. Nuclei, and spirochetes stained with propidium iodide (red), and anti-*B. burgdorferi* (N40) IgG (FITC-green) respectively. In **A** and **C** magnification ×40. **D**. Spirochetes in each salivary gland pair counted manually. Each data point represents one salivary gland pair. **E**. Confocal microscopy of guts from 72 h fed *B. burgdorferi*-infected nymphs injected with ds *ixofin3D* or ds *gfp* RNA washed to remove unbound *Borrelia*, and PFA fixed prior to staining. Nuclei were stained with propidium iodide (red), and spirochetes with anti-*B. burgdorferi* (N40) IgG (FITC-green). Magnification ×40. **F**. qRT-PCR assessment of *Borrelia* burden in washed tick guts. Each data point in B represents a pool of 3 guts. **G**. Mean pixel intensities of regions of interest in the FITC channel (representing anti-*B. burgdorferi* serum binding) of the confocal images obtained in **E**. Each data point represents one region of interest. Error bars in **B**, **D**, **F** and **G** represent mean ± SEM. Mean values significantly different in a two-tailed non-parametric Mann-Whitney test indicated by one asterisk (*p*<0.05) or indicated by two asterisks (*p*<0.01).

*Borrelia* replicates in the gut in preparation for transmission, and adheres tightly to the gut epithelium in order to migrate towards the basal lamina of the gut epithelium, moving away from the lumen [13]. RNAi-mediated decrease in *ixofin3D* expression did not demonstrate alteration in *Borrelia* burden in the tick gut as seen by qRT-PCR (Fig. 5B). However, this assessment cannot distinguish gut epithelium-bound *Borrelia* from those that are not bound to the gut epithelium. Therefore, we assessed by confocal microscopy, if Ixofin3D might enhance *Borrelia* adherence to the gut epithelium. RNAi-mediated decrease in *ixofin3D* expression resulted in significantly decreased *Borrelia* clustering to the gut epithelium as seen by confocal microscopy (Fig. 7A) and quantification of the pixel intensity in the FITC channel (representing binding of anti-*B. burgdorferi* serum to spirochetes) using the ImajeJ software (Fig. 7B). Consistent with the qRT-PCR observations, the numbers of spirochetes was also significantly reduced in the salivary glands of ds *ixofin3D* RNA-injected nymphs (Fig. 7C–D). To further assess if Ixofin3D might facilitate spirochete aggregation to the tick gut, fed tick guts were washed to remove luminal blood-meal contents and spirochetes loosely adhering to the gut epithelium. *Borrelia* burden assessed in the washed gut epithelium by confocal microscopy and quantification of the pixel intensity in the FITC channel using the ImageJ software and by qRT-PCR showed decreased gut-bound *Borrelia* burden in ds *ixofin3D* RNA-injected nymphal guts when compared to that in ds *gfp* RNA-injected nymphal guts (Fig. 7E–G).

## Discussion

Understanding the biology of *B. burgdorferi* and its pivotal interactions with the host and the vector remains a key area of *B. burgdorferi* research [24]. In this study we focused on the tick gut, a tissue central to spirochete growth and transmission [11], [25], [26]. Little is known of the molecular interactions between the tick gut and the spirochete that facilitate exit from the gut for transmission to occur. We utilized a yeast surface display (YSD) approach and identified four tick gut proteins that might engage with the spirochete during transmission (Table 1). Zhang *et al* [14] showed that BBE31, a *Borrelia* outer surface protein binds to TRE31, a secreted tick gut protein. Our initial screening of the YSD library with *in vitro*-grown spirochetes did not identify TRE31, presumably, due to the very low expression levels of BBE31 in *in vitro* grown spirochetes [14].

Clone 1, the most frequently identified clone in this YSD screen, encoded a partial fragment of the gene ISCW008121. Three paralogs of ISCW008121 are represented in the *Ixodes scapularis* genome. BLAST analysis did not reveal orthologs of ISCW008121 in other tick species. The full-length ∼66 kDa protein referred to as Ixofin3D contained four putative fibronectin type III domains. The fibronectin type III domain was originally identified within the protein fibronectin [27]. *B. burgdorferi* encodes at least two proteins that bind to fibronectin, RevA [28] and BBK32 [29], [30], and this binding has been invoked in the infection of the murine host, but not the tick. ELISA assessment did not demonstrate the binding of RevA or BBK32 to Ixofin3D-PF (data not shown), suggesting that Ixofin3D might not be a fibronectin-like protein, and that the fibronectin III domains might provide a novel function that remain to be elucidated.

Although active and passive immunizations against rIxofin3D-PF showed a consistent trend towards decreased spirochete burden in the salivary glands, the decrease was not statistically significant. However, we observed a significant decrease in the skin seven days post-tick challenge upon active immunization against Ixofin3D. We expect that active immunization achieved higher levels of circulating antibodies and likely provided more efficient impairment of spirochete migration from the gut to the salivary glands when compared to that observed upon passive immunization. While a threshold of spirochete numbers critical for effective tick transmission is not defined, when lesser numbers of spirochetes are deposited in the skin they might be more vulnerable to the host immune responses. Similarly, RNAi-mediated silencing of *ixofin3D* expression provided a significant decrease in spirochete burden in salivary glands and consequently significantly decreased spirochete burden in the murine host skin at 7 and 14 days post-feeding. However, burden in the distal organs assessed 21 days post feeding was not different upon active immunization or RNAi-mediated knockdown of *ixofin3D* expression. This suggests that spirochetes that escape the initial host immune response, replicate, and disseminate successfully with time. Tick challenge experiments described herein utilized 4–5 *Borrelia*-infected ticks, which provides a combined inoculum of immunomodulatory tick proteins and spirochetes from 4–5 ticks, and thus potentially deflates the significance of Ixofin3D in spirochete transmission. Challenge experiments using smaller numbers of ticks might be more reflective of tick bites on humans and could be viable in studies assessing the vaccine potential of tick and *Borrelia* antigens.

Immunization or RNAi-mediated interruption of Ixofin3D-*Borrelia* interaction decreased spirochete burden in the salivary glands without any significant change in the *Borrelia* burden in the tick gut suggesting that Ixofin3D-spirochete interaction might facilitate spirochete entry into salivary glands or exit from the gut. Ixofin3D is not a secreted protein and is expressed preferentially in the gut, and RNAi-mediated decrease in Ixofin3D was specific to the gut. Therefore, Ixofin3D is more likely to provide a function to the spirochete in the gut. Work by Dunham-Ems [13] has shown that spirochetes migrate through the gut as sheets of spirochete aggregates. RNAi-mediated decrease in *ixofin3D* resulted in decreased gut epithelium-bound spirochetes. The aggregation of spirochetes on the tick gut might provide critical signals essential for spirochete migration through the gut. Coincident with tick feeding, there is a large increase in spirochete numbers [26] and residence in the luminal space would not be conducive to transmission [13]. Ixofin3D might serve as a sticky mat to facilitate spirochete congregation to the gut epithelium. The clustering of spirochetes to Ixofin3D on the tick gut might provide a molecular direction to aid spirochete exit from the gut.

Ixofin3D is expressed in uninfected nymphal ticks fed on uninfected mice, and likely serves a physiological function in the tick gut. Although tick feeding was not altered upon immunization against rIxofin3D-PF or RNAi-mediated decrease in *ixofin3D* expression, we cannot rule out the possibility that Ixofin3D might play a role in gut functions unrelated to feeding efficiency, and this might also modulate spirochete-gut interactions critical for transmission. The observation that Ixofin3D expression was significantly increased in *B. burgdorferi*-infected tick guts suggested that a specific spirochete ligand might be responsible for this increase, or it might represent a tick gut response to the spirochete. In future efforts, successful identification of the *Borrelia* surface protein that binds to Ixofin3D might illuminate a mechanistic understanding of Ixofin3D and its interaction with *Borrelia*. This study provides a new insight into tick-spirochete interactions in the gut and offers a molecular handle to unravel the biological significance of spirochete aggregation and the functional consequence on egress from the gut. Exit from the tick gut is fundamental to transmission and a molecular understanding of this event could provide new targets to prevent *Borrelia* transmission.

## Materials and Methods

### Ethics statement

Animals were housed and handled under the Guide for the Care and Use of Laboratory Animals of the National Institutes of Health. The animal experimental protocol was approved by the Yale University's Institutional Animal Care & Use Committee (protocol number 2008-07941, approval date: 3/31/2014). All animal infection experiments were performed in a Bio-safety Level 2 animal facility, according to the regulations of Yale University.

### Ticks

*I. scapularis* nymphs and larvae were obtained from a tick colony at the Connecticut Agricultural Experiment Station in New Haven CT, USA and ticks maintained as described earlier [22].

### Yeast surface display library screening

cDNAs prepared from uninfected *I. scapularis* nymphs that were fed on uninfected C3H/HeN mice for 72 hours were directionally cloned by ligation into the *Not*I-*Eco*RI sites of the pYD1 yeast display vector (Invitrogen, Carlsbad, CA) to obtain a primary unamplified titre of 4×10^6^ CFU/ml with 98% recombination efficiency [14]. Total plasmid DNA was prepared from the primary library and transformed into EBY100 *Saccharomyces cerevisiae* strain as described by Chao et al [31] and about 1×10^7^ individual YSD clones were utilized for the screening as detailed below. *Borrelia burgderfori* N40 membrane protein extracts were purified as described by Nowalk et al [18], and biotin labeled using the EZ-Link Sulfo-NHS-Biotinylation Kit (Thermoscientific, Rockford, IL). An overnight culture of 10^8^ yeast cells were induced as detailed by Chao et al [31], washed 3 times with cold PBS, 0.5% BSA, 2 mM EDTA (MACS) buffer, and incubated with 30 µg of biotinylated *B. burgdorferi* membrane proteins for 1 h at 4°C. Cells were then washed three times and incubated with anti-biotin microbeads (Miltenyi Biotec, Auburn, CA). Cells were washed three times, resuspended in 30 ml of MACS buffer and subjected to magnetic separation to enrich for *B. burgdorferi*-membrane protein bound YSD clones as described before [32]. The magnetically sorted cells were grown in SDCAA medium for 24 hours at 30°C and *Borrelia*-interacting clones were enriched by four rounds of MidiMACS sorting under the same conditions as described above. At each round of sorting, an aliquot of the induced cells was incubated with 10 µg of Alexa-488 conjugated-*B. burgdorferi* membrane protein extracts for 1 h at 4°C, washed three times and analyzed on a FACS Calibur flow cytometer (Beckton Dickinson, Franklin Lakes, NJ) to assess binding. Induction of the YSD library and surface expression of clones was verified by indirect immunostaining with anti-Xpress-epitope [31]. Ten thousand cells were examined on a FACS Calibur flow cytometer and data analyzed using the FlowJo software (Tree Star, Ashland, OR). For screening of individual clones, individual yeast clones were grown overnight, induced and binding to Alexa-488 conjugated-*B. burgdorferi* membrane protein extracts assessed as described above. YSD clones that demonstrated 15% or more binding were selected for further analysis. Plasmid DNA isolation, insert size assessment and prioritization for sequencing was performed as described earlier [32].

### Tick RNA isolation and quantitative RT-PCR

Ticks were allowed to feed for 24 h, for 72 h, or to repletion (between 80 and 96 h after initiation of tick feeding) and RNA isolated from guts and salivary glands using Trizol (Invitrogen, CA) as described earlier [22]. cDNA was synthesized using the iScript RT-PCR kit (Bio-Rad, CA) and analyzed by quantitative PCR for the expression of tick *actin* and *B. burgdorferi* and also *ixofin3D*, clone 2, 3 and 4 using gene-specific primers (listed in table S1) and the iQ SYBR Green Supermix (Bio-Rad, Hercules, CA) on a Opticon Engine MJ cycler (Bio-Rad, CA).

### Identification of the full-length transcript of ISCW008121

The RLM-RACE kit was used to identify the sequence at the 3′-end and 5′end according to the manufacturer's instructions (Invitrogen, CA). First strand cDNA was synthesized from total *I. scapularis* gut RNA using the 3′-RACE Adapter. The cDNA was then subjected to a PCR using the outer 3′-RACE primer, which is complementary to the distal part of the anchored adapter and an Ixofin3D specific primer Ixofin3D_882FW (Table S1) complementary to Nt 882- 901. The PCR product was then subjected to a nested PCR on an inner 3′-RACE primer and an Ixofin3D specific primer complementary to Nt 975-993, Ixofin3D_975FW (Table S1). The identification of the sequence at the 5′end was performed using Ixofin3D-specific primers Ixofin3D_595RV and Ixofin3D_475RV as described before [33]. The full-length sequence was assembled using the web-based software SMART [34] (http://smart.embl-heidelberg.de).

### Purification of recombinant *I. scapularis* Ixofin3D

Ixofin3D was cloned in-frame into the pMT-Bip-V5-His tag vector (Invitrogen, CA) using ixofin3D_DESF and ixofin3D_DESR primers listed in Table S1 and recombinant protein expressed and purified using the *Drosophila* Expression System (Invitrogen, CA) as described earlier [22]. Recombinant protein purity was assessed by SDS-PAGE, and quantified using the BCA protein estimation kit (Thermoscientific, IL).

### *In vitro* binding of Ixofin3D to *B. burgdorferi*

*B. burgdorferi* (strain N40) was cultured at 33°C and washed 3 times with PBS, resuspended in 500 µl PBS at 10^5^ spirochetes/500 µl and fixed with 4% PFA for 20 min at RT and washed 3 times with PBS. Spirochetes were blocked with 5% FCS in PBS for 1 hour followed by incubation overnight with 1 µg of rIxofin3D or rIxophilin, a tick gut thrombin inhibitor [22], at 4°C. After 3 washes in PBS, spirochetes were incubated with purified polyclonal rabbit IgG against rIxofin3D-PF or polyclonal mouse IgG against rIxofilin, washed 3 times and incubated with 1∶2000 diluted anti-rabbit or anti-mouse TRITC respectively. The spirochetes were then washed 3 times and stained with FITC-conjugated goat anti *B. burgdorferi* antibodies (KPL, MD) for 1 hour, washed and mounted in Antifade Gold (Biorad, CA) and visualized under a fluorescence microscope (Axiovert 200M; Zeiss, Jena, Germany) at 20× magnification.

### ELISA assessment of rIxofin3D-PF binding to *B. burgdorferi* membrane extract

*B. burgdorferi* membrane extract purified as described above was coated (1 µg/ml) on high binding microtiter plates (Microlon, Greiner, Germany) overnight at RT. Wells were blocked with PBS/1% BSA at RT for 1 h and incubated with rIxofin3D-PF or rIxophilin (3–100 pmol/ml) diluted in PBS/0.05%Tween20/1% BSA for 1 h. Wells were washed and incubated with 1∶500 diluted rabbit anti rIxofin3D or mouse anti-Ixophilin IgG and bound antibody detected using HRP-conjugated anti-rabbit and anti-mouse antibody respectively (Sigma, MO) and TMB as substrate (Thermoscientific, IL).

### Passive or active immunization of mice against rIxofin3D-PF and assessment of *B. burgdorferi* transmission

New Zealand white rabbits 4–6 weeks old were immunized subcutaneously with 30 µg of rIxofin3D-PF or ovalbumin in complete Freund's adjuvant and boosted twice with 30 µg of rIxofin3D-PF or ovalbumin once every 3 weeks in incomplete Freund's adjuvant. Test bleeds were obtained from ear veins 2 weeks after the final boost and reactivity to recombinant rIxofin3D-PF and ovalbumin assessed by western blot. Rabbits were euthanized and serum was obtained by cardiac puncture. Polyclonal IgG was purified from the sera using the Melon Gel IgG purification kit (Thermoscientific, IL). For passive immunization, mice were passively immunized 24 h prior to tick placement by intraperitoneal inoculation with 100 µg of purified rabbit IgG against rIxofin3D-PF or ovalbumin. For active immunization, mice were immunized with 10 µg of rIxofin3D-PF or ovalbumin as described for rabbits. To address the role of rIxofin3D-PF in *B. burgdorferi* transmission, four *B. burgdorferi* N40 infected nymphs were placed on each immunized mouse. Nymphs were allowed to feed to repletion. Salivary glands and guts were dissected and combined in pools of 2–3 ticks for quantitative RT-PCR as described above. DNA was isolated from skin punch-biopsies at 7, 14 and 21 days and from heart and joints 21 days post tick-detachment and *Borrelia* burden assessed by quantitative PCR as described [35].

### RNAi silencing of *ixofin3D* in *Borrelia*-infected *I. scapularis* nymphs

RNAi silencing of *ixofin3D* in ticks was performed as described before [35] using primers, specific for *ixofin3D* with an T7 promoter sequence, Ixofin3D_dsRNAFW and Ixofin3D_dsRNARV (Table S1). ds ixophin3D dsRNA was synthesized using the MEGAscript RNAi kit (Ambion/Invitrogen, CA). ds ixophin3D RNA or ds gfp RNA (5 nl, 3×10^12^ molecules/ml) was injected into the anal pore of *Borrelia*-infected nymphs as described earlier [35]. dsRNA-injected ticks were allowed to feed until repletion and weighed to assess feeding efficiency, and guts and salivary glands dissected for mRNA isolation and quantitative RT-PCR as described above. *B. burgdorferi* burden in mice was assessed by quantitative PCR as described earlier [35].

### Confocal microscopy

Guts from nymphal ticks (*B. burgdorferi*-infected or uninfected) were dissected and fixed in 4% PFA for 20 minutes, washed in PBS/0.5% Tween20 (three times) and blocked in PBS/0.5%Tween20, 5% fetal calf serum prior to sequential incubation with rabbit anti-*B. burgdorferi* N40 antibody and bound antibodies detected using FITC-labeled affinity purified goat anti rabbit IgG antibody (Sigma, MO) and nuclei stained with propidium idodide or with TOPRO-3 iodide (Invitrogen, CA). In experiments where Ixofin3D were visualized, tick guts were fixed as described above incubated with IgG purified from rabbit anti-Ixofin3D-PF sera. Control guts were incubated with Ig purified from rabbit anti-ovalbumin sera. Bound antibodies were detected using TRITC-labeled affinity purified mouse anti rabbit IgG antibody (Sigma, MO). All incubations were conducted in moist chambers at room temperature for 1 hour. All washes were done 3 times in PBS. Stained guts were visualized under a Zeiss LSM510 Confocal microscope.

### Pixel intensity quantification

Pixel intensities in the TRITC channel (as a measure of anti-Ixofin3D-PF serum binding to tick gut Ixofin3D) or in the FITC channel (as a measure of anti-*B. burgdorferi* serum binding to spirochetes) of confocal images were quantified using ImageJ 1.47t software available in the public domain (http://imagej.nih.gov/ij). Confocal images of 5–6 individual guts were examined in each control and experimental group and mean pixel intensities representing the average intensity of pixels in the region of interest were obtained in at least 5 different regions of each tick gut.

### Separation of gut epithelium away from gut luminal contents

Nymphal ticks fed for 72 h were carefully dissected and the tips of each gut diverticulum nicked with a razor blade to let the luminal contents discharge out, placed in 500 µl of cold PBS and allowed to stand for 5 minutes. The supernatant was aspirated and PBS wash repeated three more times. The guts were then fixed in PFA as described above for confocal microscopy to visualize spirochetes using rabbit anti-*B. burgdorferi* (N40) antisera or suspended in Trizol for RNA preparation as described above.

### Bioinformatic tools for *in silico* analysis of DNA and protein

DNA sequences obtained by Sanger sequencing were trimmed and translated to protein sequence using the Lasergene 7 DNA analysis tool (DNASTAR Inc, WI) and homology to DNA and protein sequences in the NCBI database determined by BLAST analysis [36]. Assembly of DNA sequences obtained by 5′ and 3′ RACE was performed using CodonCode Aligner 4.2.3. Predicted proteins encoded by the *Borrelia*-interacting YSD clones were analysed for the presence of secretory signal sequences using Signal P4.1 software (www.cbs.dtu.dk/services/SignalP), cellular localization assessed using the PSORT software (http://wolfpsort.org), protein domains using the Simple Modular Architecture Research Tool available at http://smart.embl-heidelberg.de and theoretical molecular weight (MW) and isoelectric point (pI) using ExPASy proteomics server (http://web.expasy.org/compute_pi/).

### Statistical analysis

The significance of the difference between the mean values of the groups was analyzed using a non-parametric two-tailed Mann-Whitney test or a two-tailed student *t* test with Prism 5.0 software (GraphPad Software, San Diego, CA), and p<0.05 was considered significant. One-way ANOVA (Analysis of Variance) with Tukey's multiple comparison test was utilized when the mean values of more than 2 groups were compared.

### Accession numbers

The GenBank accession numbers and VectorBase accession numbers for genes/proteins related with this study: TROSPA: AY189148.1, Tre31: HQ998856, BBE31: NP_045436.1, Clone1: ISCW008121, Ixofin-3D: KF709698. Clone2: ISCW015135, Clone3: ISCW015049, Clone4: ISCW016197, RevA (BBM27): NP_051318.1, BBK32: AAL84596.1, Ixophilin: ISCW003862.

## Supporting Information

Table S1 **Primers utilized in this study.**
(DOCX)
