# Supplementary material for: A Tick Gut Protein with Fibronectin III Domains Aids Borrelia burgdorferi Congregation to the Gut during Transmission
Source: PLoS Pathog. 2014 Aug 7;10(8):e1004278. doi: 10.1371/journal.ppat.1004278 (PMC4125277; doi:10.1371/journal.ppat.1004278)
Supplement: Table S1 — Primers utilized in this study. (DOCX) [file ppat.1004278.s001.docx]

**Table S1. Primers utilized in this study**

| **Primer name** | **Primer sequence** | **Function** |
| --- | --- | --- |
| Ixofin3D_882FW | GGCTACACGGTGTTCTACGG | 3’ END RLM RACE |
| Ixofin3D_975FW | GCCAACACAGAGTTCGAGGT | 3’ END RLM RACE |
| Ixofin3D_595RV | CGGAAGTCGGTCAGAGTCC | 5’ END RLM RACE |
| Ixofin3D_475RV | GTGACCGGGCCCACGCCTCC | 5’ END RLM RACE |
| Ixofin3D_486FW | CGGTCACCTGTCAGCACAT | *ixofin3D* sequencing |
| Ixofin3D_1551RV | CACGACGCTCACGTTGTACT | *ixofin3D* sequencing |
| Ixofin3D_RTFW | GCCAACACAGAGTTCGAGGT | *ixofin3D* expression |
| Ixofin3D_RTRV | CCACAGAGTTCTTGCTCGTG | *ixofin3D* expression |
| Clone2_RTFW | CTGCAGTGTCCGCCGGAGAGTGA | Clone 2 expression |
| Clone2_RTRV | AGCAAAACGGGAAACAAG | Clone 2 expression |
| Clone3_RTFW | AGGACTCTGCATGGACAAGG | Clone 3 expression |
| Clone3_RTRV | AGCAGCATCACTGCAATCAC | Clone 3 expression |
| Ixofin3D_DESFW | CCATGGTGCCAGACGTCCAAC | Recombinant Ixofin3D-PF |
| Ixofin3D_DESRV | CTCGAGCGAGCTCGTTGAAGG | Recombinant Ixofin3D-PF |
| Ixofin3D_dsRNAFW | TAATACGACTCACTATAGGGAGAAGAAGCACCGGGAGAAGAGC | dsRNA *ixofin3D* |
| Ixofin3D_dsRNARV | TAATACGACTCACTATAGGGAGATCGACCACCACAGAGTTCTTGC | dsRNA *ixofin3D* |
